# Supplementary material for: Myc plays an important role in Drosophila P-M hybrid dysgenesis to eliminate germline cells with genetic damage
Source: Commun Biol. 2020 Apr 22;3:185. doi: 10.1038/s42003-020-0923-3 (PMC7176646; doi:10.1038/s42003-020-0923-3)
Supplement: Supplementary file 1 — Supplementary Information [file 42003_2020_923_MOESM1_ESM.pdf]

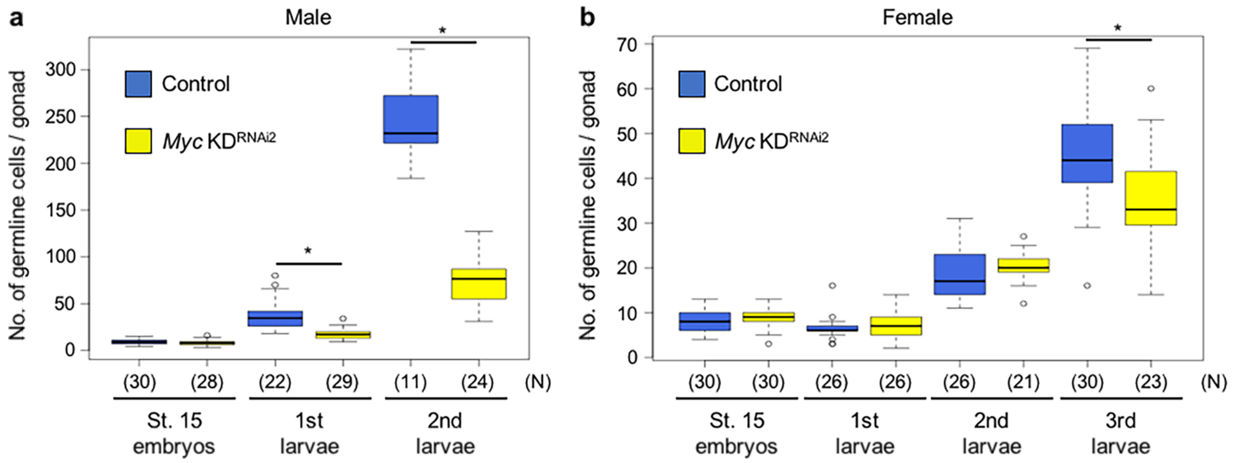

Supplementary Figure 1: Reduction of germline cell number in *Myc* knockdown using a second *Myc* RNAi line.
(a and b) The number of germline cells of male (a) and female (b) progeny derived from *nos-Gal4*/*nos-Gal4* females mated with *nos-Gal4*/*nos-Gal4* (Control; blue) and *UASt-Myc*^{RNAi2}/*UASt-Myc*^{RNAi2} [*Myc* KD^{RNAi2} (BDSC51454); yellow] males at early embryonic stage 15 and early-first, second, and third instar stages. Gonads were stained for Vasa, and Vasa-positive germline cells in gonads were counted. Each box plot represents median value and first and third quartile values. Error bars represent minimum and maximum values. White circles represent outliers. Significance was calculated by two-sided Student's t-test; *, $P < 0.05$. N: total number of the observed gonads. Similar results were obtained from two biologically independent experiments.

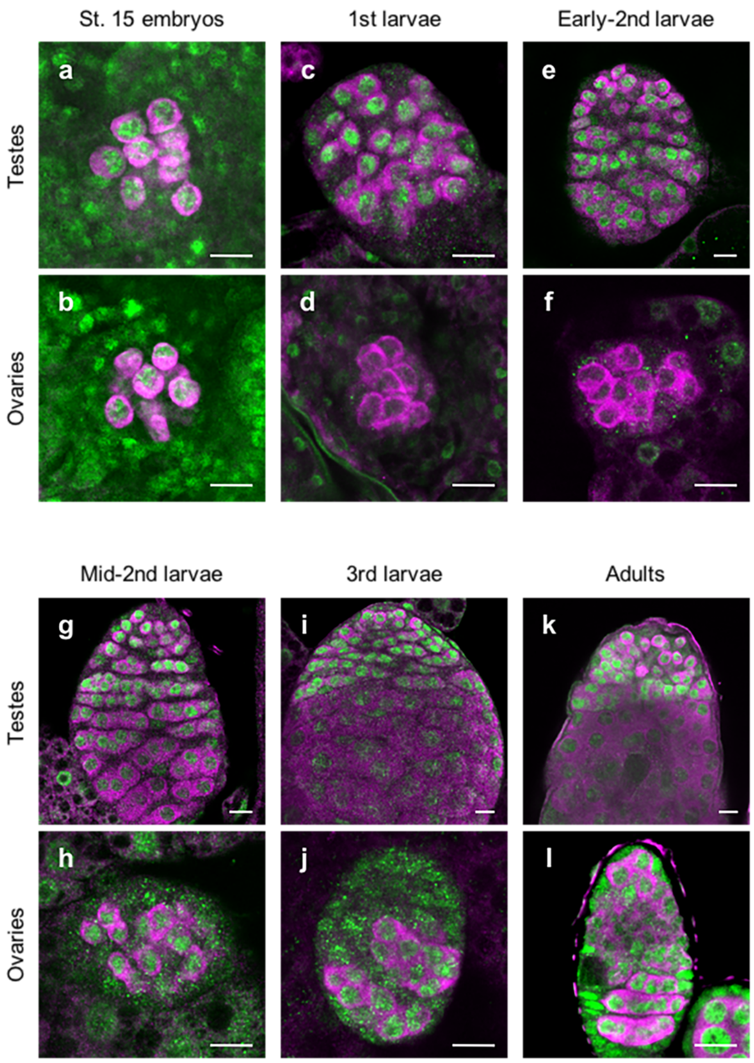

Supplementary Figure 2: Expression of Myc-GFP during germline development.
Expression of Myc-GFP in testes (a, c, e, g, i, and k) and ovaries (b, d, f, h, j, and l) of early stage-15 embryos (a and b); early-first instar (c and d), early-second instar (e and f), mid-second instar (g and h), early-third instar (i and j) larvae; and adults 3–5 days after eclosion (k and l). All embryos, larvae, and adults were of the *Myc-GFP*/*Myc-GFP* line. Gonads were stained for GFP (green) and Vasa (magenta). Distal regions of adult testis and ovary are shown in k and l, respectively. Scale bars: 10 µm.

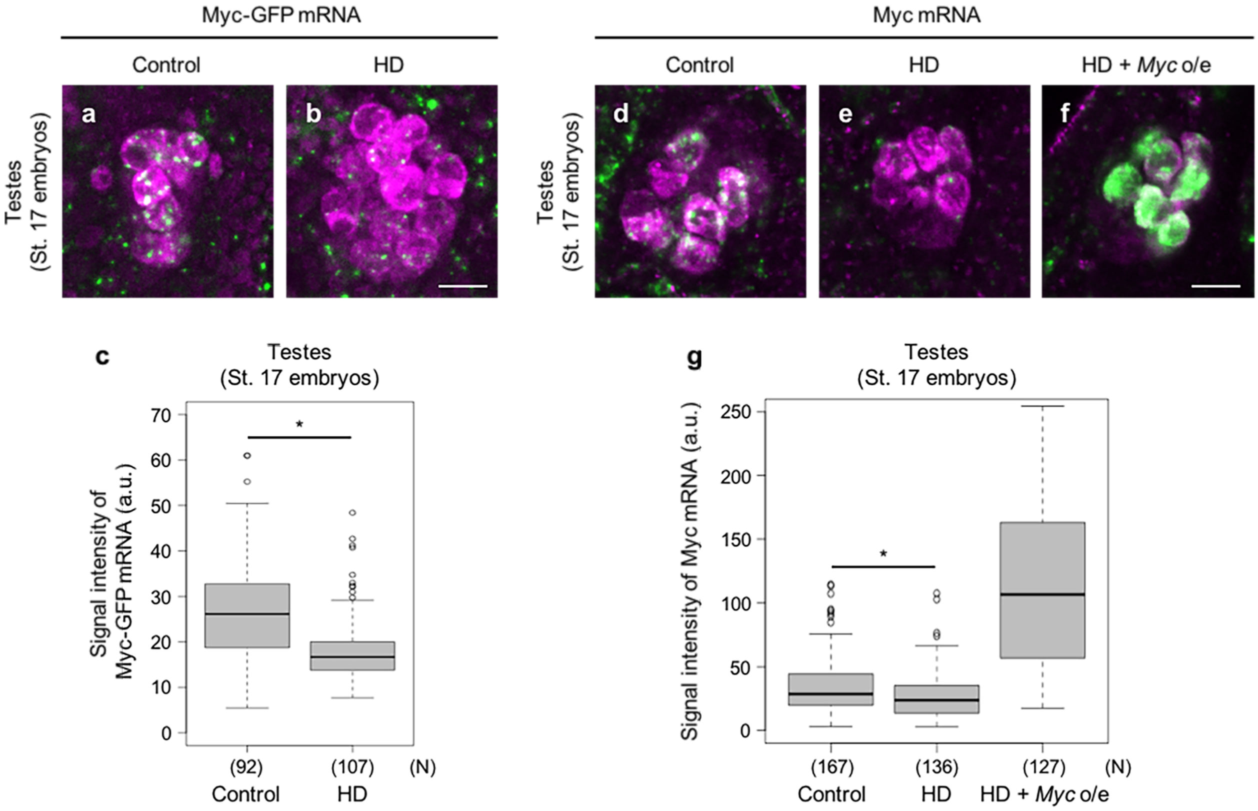

Supplementary Figure 3: Myc-GFP and Myc mRNA expression in HD germline.
(a and b) Myc-GFP mRNA expression in the germline cells of male derived from *Myc-GFP*/*Myc-GFP* females mated with *y w* (Control; a) and *Harwich* (HD; b) males at embryonic stage 17. Gonads were stained for Myc-GFP mRNA (green) and Vasa (magenta). Scale bar: 10 µm. (c) Signal intensity of Myc-GFP mRNA in germline cell of Control and HD at embryonic stage 17. Each box plot represents median value and first and third quartile values. Error bars represent minimum and maximum values. White circles represent outliers. Significance was calculated by two-sided Student's t-test; *, $P < 0.05$. N: total number of the observed gonads. Similar results were obtained from two biologically independent experiments. (d–f) Myc mRNA expression in the germline cells of male derived from *nos-Gal4*/*nos-Gal4* females mated with *nos-Gal4*/*nos-Gal4* males (Control; d), *nos-Gal4*/*nos-Gal4* females mated with *Harwich* males (HD; e), and *nos-GAL4 UASt-Myc*/*TM3*, *Act5C-GFP* females mated with *Harwich* males (GFP-negative progeny were selected as HD + *Myc* o/e; f) at early embryonic stage 17. Gonads were stained for Myc mRNA (green) and Vasa (magenta). Scale bar: 10 µm. (g) Signal intensity of Myc mRNA in germline cells of Control, HD, and HD + *Myc* o/e at early embryonic stage 17. Each box plot represents median value and first and third quartile values. Error bars represent minimum and maximum values. White circles represent outliers. Significance was calculated by two-sided Student's t-test; *, $P < 0.05$. N: total number of the observed gonads. Similar results were obtained from two biologically independent experiments.

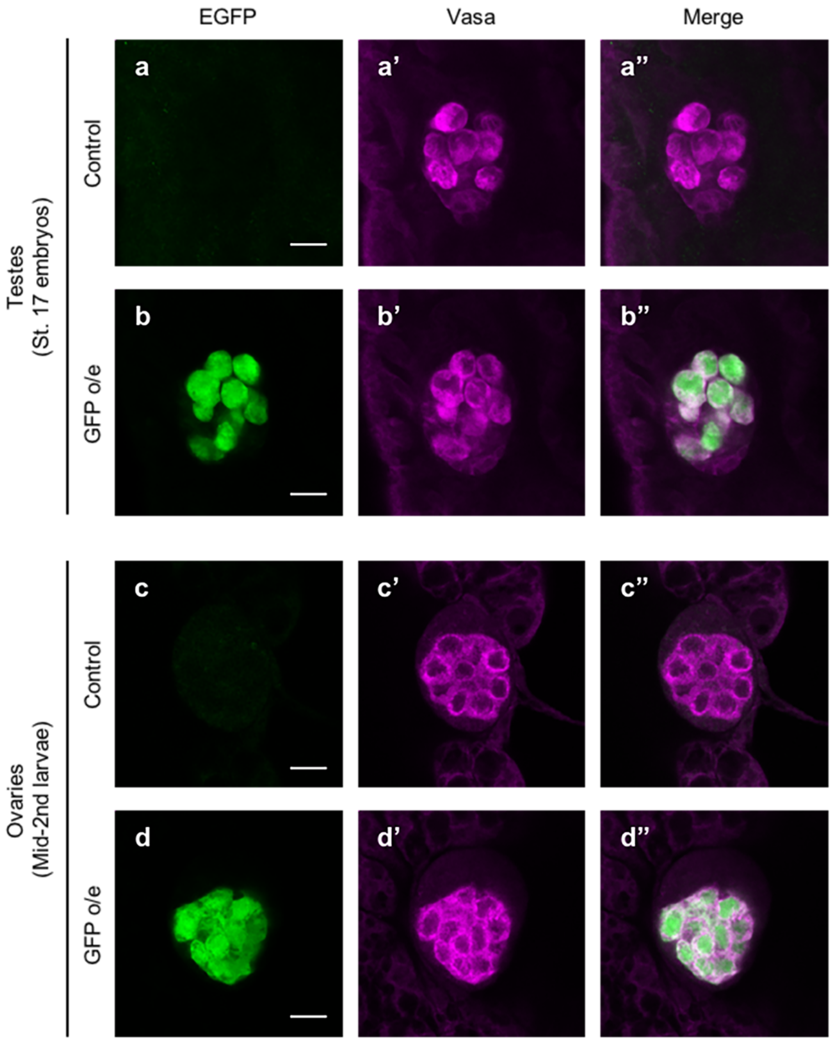

Supplementary Figure 4: *UASt-EGFP* expression in the germline under the control of *nos-Gal4*.
EGFP expression in the germline of testes at the embryonic stage 17 (a and b) and ovaries at the mid-second instar stage (c and d). Gonads in progeny derived from *nos-Gal4*/*nos-Gal4* females mated with *nos-Gal4*/*nos-Gal4* (Control; a and c) and *UASt-EGFP*/*UASt-EGFP* (GFP o/e; b and d) males were stained for EGFP (green; a–d) and Vasa (magenta; a’–d’). Merged images are also indicated (a”–d”). Males and females were cultured at 29°C and 25°C, respectively. Scale bars: 10 µm.

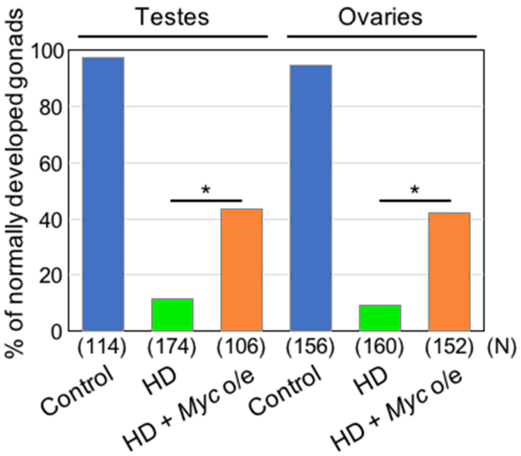

Supplementary Figure 5: Percentage of normally developed gonads in HD progeny with or without *Myc* overexpression.

Gonads of progeny derived from *nos-Gal4*/*nos-Gal4* females mated with *nos-Gal4*/*nos-Gal4* (Control), *nos-Gal4*/*nos-Gal4* females mated with *Harwich* males (HD), and *nos-GAL4 UASt-Myc*/*TM3*, *Ser Sb* females mated with *Harwich* males (non-*Ser* and non-*Sb* progeny were selected as HD + *Myc* o/e) were obtained from adults 3–5 days after eclosion, and their morphologies were observed. Ovaries with more than three mature eggs and testes > 1 mm in length were considered normally developed gonads. Significance was calculated by two-sided Fisher's exact test; *, $P < 0.05$. N: total number of the observed gonads. Similar results were obtained from two biologically independent experiments.

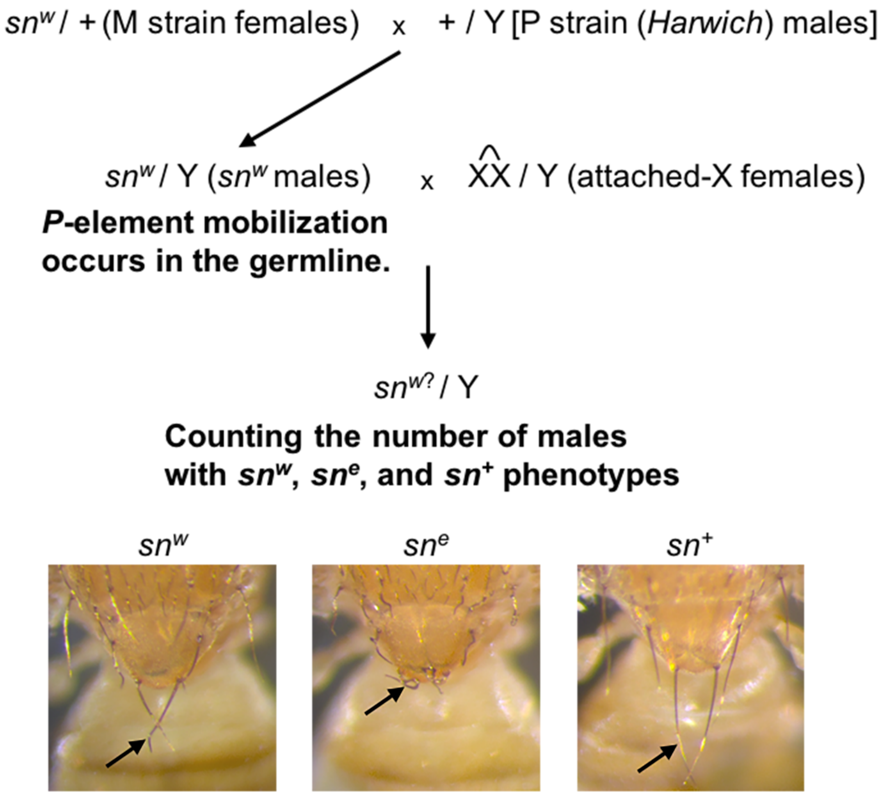

Supplementary Figure 6: Schematic diagram of mating scheme for examining sn^w-mutation frequency in the germline.

P-element mobilization in the germline of $singed^{weak}$ male progeny (sn^w/Y) derived from sn^w/+ females mated with *Harwich* males results in either a wild-type (sn^+) or extreme *singed* (sn^e) phenotype in male offspring derived from the sn^w/Y males mated with attached-X females (XX/Y). sn^w-mutation frequency was determined by counting the male offspring with sn^w, sn^e, and *sn*+ phenotypes. Arrows indicate apical scutellar bristles.

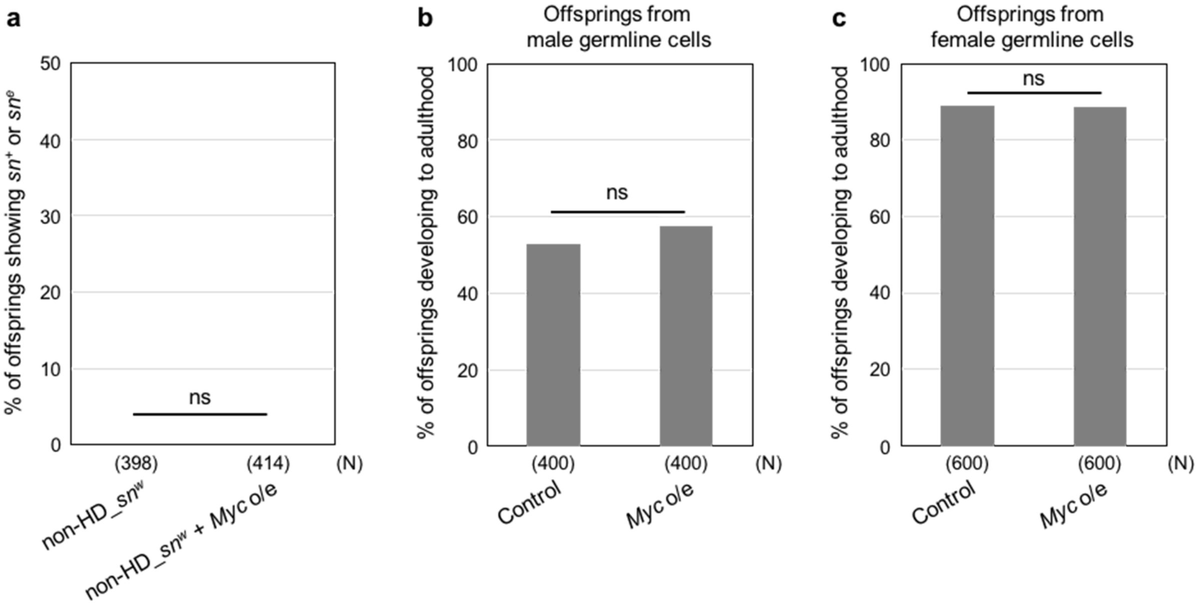

Supplementary Figure 7: Effects of *Myc* overexpression in non-HD germline.
(a) Mutation frequency in the germline was examined using the sn^w allele. The mating scheme is shown in Supplementary Table 3. non-HD_sn^w and non-HD_sn^w + *Myc* o/e were mated with attached-X females. Percentages of male offspring with sn^e and sn^+ phenotypes are shown. No sn^e or sn^+ male offspring were produced by either non-HD_sn^w or non-HD_sn^w + *Myc* o/e males. Significance was calculated by two-sided Fisher's exact test; ns, not significant ($P \geq 0.05$). N: total number of the observed offspring. Similar results were obtained from two biologically independent experiments. (b and c) Percentage of offspring that developed to adulthood. Offspring were produced from Control and *Myc* o/e males (b) and females (c) mated with *y w* females and males, respectively. Mating scheme is shown in Supplementary Table 4. Significance was calculated by two-sided Fisher's exact test; ns, not significant ($P \geq 0.05$). N: total number of the observed offspring. Similar results were obtained from two biologically independent experiments.

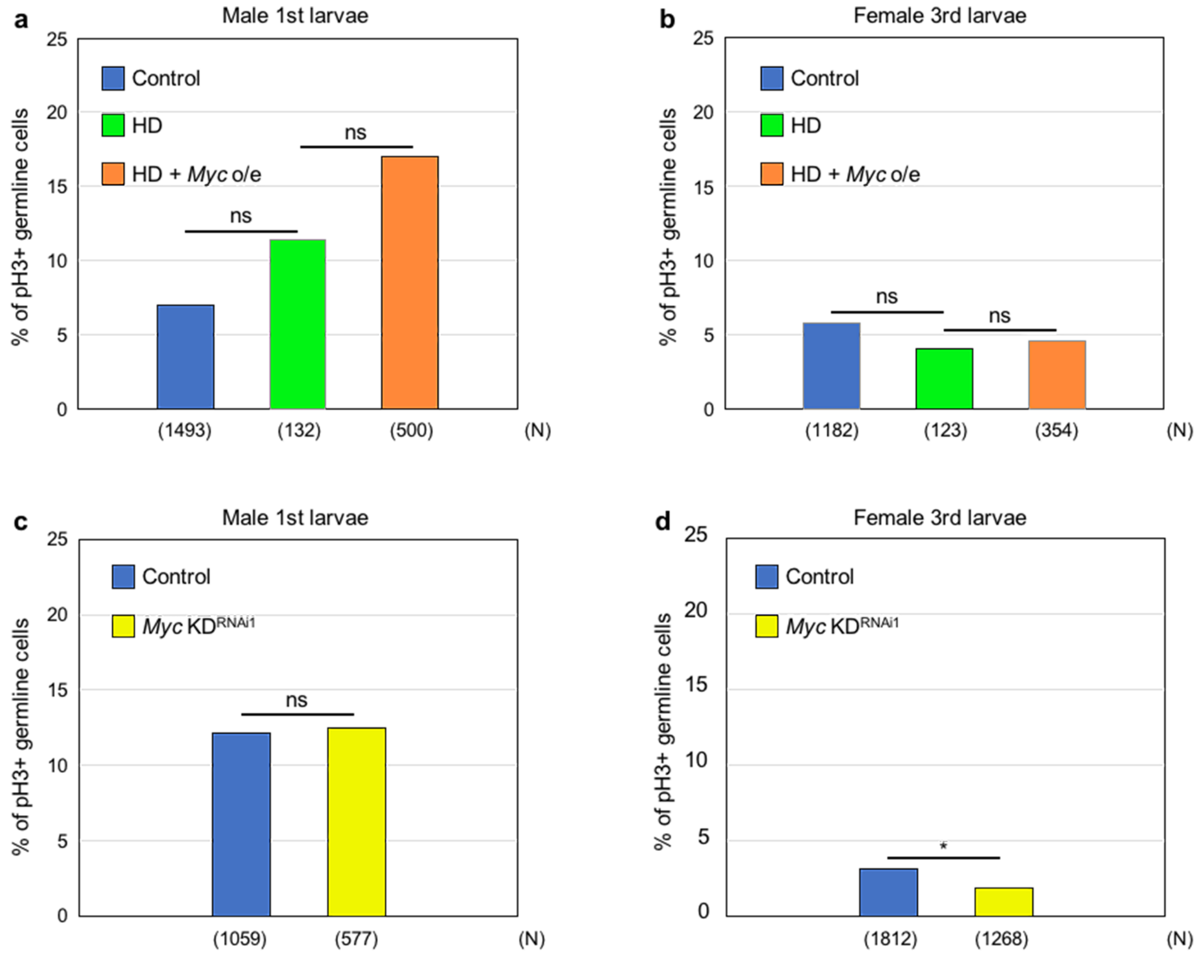

Supplementary Figure 8: Percentage of pH3-positive germline cells in HD progeny with or without *Myc* overexpression and in *Myc* knockdown.

(a and b) Percentage of pH3-positive germline cells in gonads of early-first instar male (a) and early-third instar female (b) progeny derived from *nos-Gal4*/*nos-Gal4* females mated with *nos-Gal4*/*nos-Gal4* males (Control; blue), *nos-Gal4*/*nos-Gal4* females mated with *Harwich* males (HD; green), and *nos-GAL4*, *UASt-Myc*/*TM3*, *Act5C-GFP* females mated with *Harwich* males (GFP-negative progeny were selected as HD + *Myc* o/e; orange). Gonads were stained for pH3 and Vasa, and pH3-positive germline cells in gonads were counted. Significance was calculated by two-sided Fisher's exact test; ns, not significant ($P \geq 0.05$). N: total number of the observed gonads. Similar results were obtained from two biologically independent experiments. (c and d) Percentage of pH3-positive germline cells in gonads of early-first instar male (c) and early-third instar female (d) progeny derived from *nos-Gal4*/*nos-Gal4* females mated with *nos-Gal4*/*nos-Gal4* (Control; blue) and *UASt-Myc*^{RNAi1}/*UASt-Myc*^{RNAi1} (*Myc* KD^{RNAi1}; yellow) males. Gonads were stained for pH3 and Vasa, and pH3-positive germline cells in gonads were counted. Significance was calculated by two-sided Fisher's exact test; *, $P < 0.05$; ns, not significant ($P \geq 0.05$). N: total number of the observed germline cells. We performed two biologically independent experiments. The data combining these experimental results are shown.

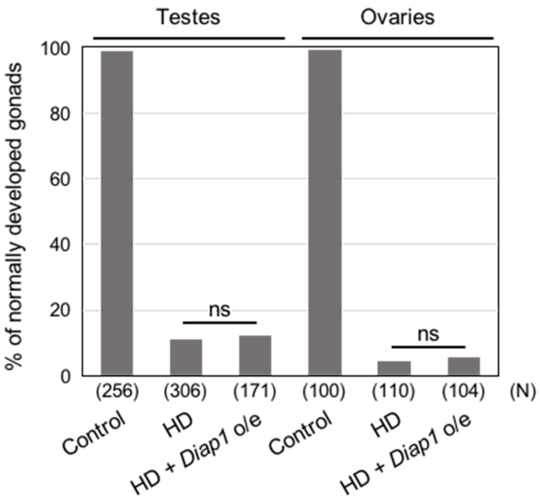

Supplementary Figure 9: Overexpression of *Diap1* does not rescue the germline-loss phenotype caused by HD.
Percentage of normally developed gonads in progeny derived from *nos-Gal4*/*nos-Gal4* females mated with *nos-Gal4*/*nos-Gal4* males (Control), *nos-Gal4*/*nos-Gal4* females mated with *Harwich* males (HD), and *nos-GAL4*, *UASt-Diap1*/*nos-GAL4*, *UASt-Diap1* females mated with *Harwich* males (HD + *Diap1* o/e). Gonads were obtained from adults 3–5 days after eclosion, and their morphologies were observed. Ovaries with more than three mature eggs and testes >1 mm in length were considered normally developed gonads. Significance was calculated by two-sided Fisher's exact test; ns, not significant ($P \geq 0.05$). N: total number of the observed gonads. Similar results were obtained from two biologically independent experiments.

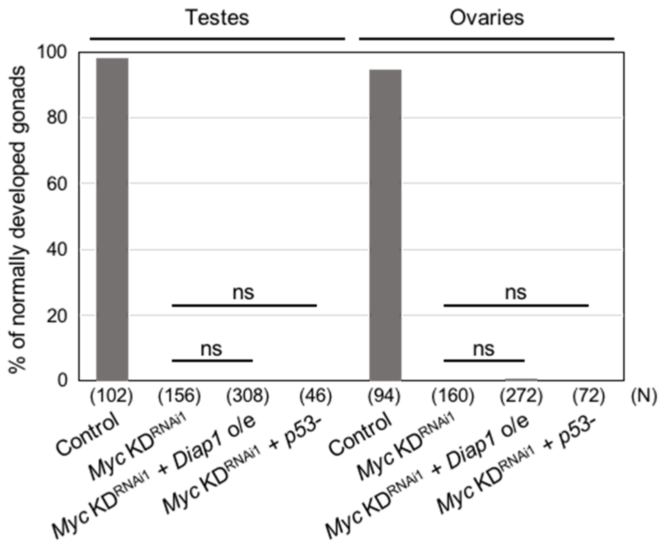

Supplementary Figure 10: Neither *Diap1* overexpression nor *p53* knockdown rescues germline-loss phenotype caused by *Myc* knockdown.

Percentage of normally developed gonads of progeny derived from *nos-Gal4*/*nos-Gal4* females mated with *nos-Gal4*/*nos-Gal4* males (Control), *nos-Gal4*/*nos-Gal4* females mated with *UASt-Myc*^{RNAi1}/*UASt-Myc*^{RNAi1} males (*Myc* KD^{RNAi1}), *nos-GAL4*, *UASt-Diap1*/*nos-GAL4*, *UASt-Diap1* females mated with *UASt-Myc*^{RNAi1}/*UASt-Myc*^{RNAi1} males (*Myc* KD^{RNAi1} + *Diap1* o/e), and *nos-GAL4*, *p53*$^{5A\text{-}4\text{-}1}$/*nos-GAL4*, *p53*$^{5A\text{-}4\text{-}1}$ females mated with *UASt-Myc*^{RNAi1}, *p53*$^{5A\text{-}4\text{-}1}$/*UASt-Myc*^{RNAi1}, *p53*$^{5A\text{-}4\text{-}1}$ males (*Myc* KD^{RNAi1} + *p53*-). Because *p53*$^{5A\text{-}4\text{-}1}$ mutation could not be introduced into *Harwich* males, an inducer of HD, its effect on the phenotype caused by HD remains elusive. Gonads were obtained from adults 3–5 days after eclosion, and their morphologies were observed. Ovaries with more than three mature eggs and testes > 1 mm in length were considered normally developed gonads. Significance was calculated by two-sided Fisher's exact test; ns, not significant ($P \geq 0.05$). N: total number of the observed gonads. We performed two biologically independent experiments for control, *Myc* KD^{RNAi1}, and *Myc* KD^{RNAi1} + *Diap1 o/e*, and obtained similar results. The data for *Myc* KD^{RNAi1} + *p53*- was obtained from a single experiment, due to a failure in maintaining *Myc* KD^{RNAi1} + *p53*- line.

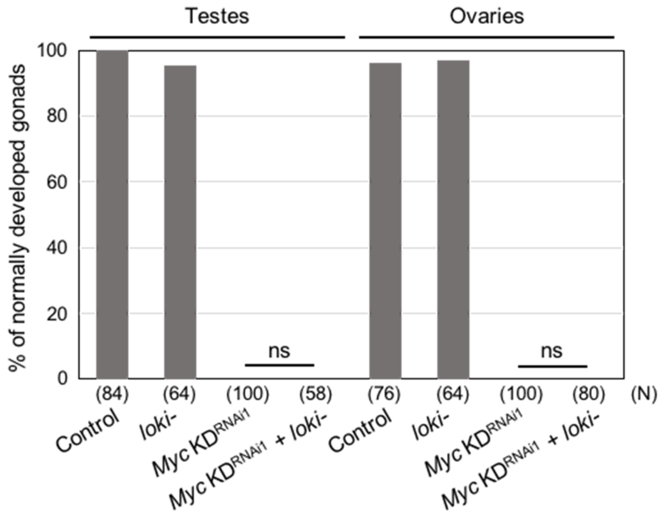

Supplementary Figure 11: Loss of function of *loki* (Chk2) does not rescue germline-loss phenotype caused by *Myc* knockdown.
Percentage of normally developed gonads in progeny derived from *nos-Gal4*/*nos-Gal4* females mated with *nos-Gal4*/*nos-Gal4* males (Control), $loki^{P6}/loki^{P6}$ females mated with $loki^{P6}/loki^{P6}$ males (*loki*-), *nos-Gal4*/*nos-Gal4* females mated with $UASt\text{-}Myc^{RNAi1}/UASt\text{-}Myc^{RNAi1}$ males (*Myc* KD^{RNAi1}), and $loki^{P6}/Cyo$; *nos-Gal4*/*TM3*, *Ser Sb* females mated with $loki^{P6}/Cyo$; $UASt\text{-}Myc^{RNAi1}/TM3$, *Ser Sb* males (non-*Cyo*, non-*Ser*, and non-*Sb* progeny were selected as *Myc* KD^{RNAi1} + *loki*-). Because the $loki^{P6}$ mutation could not be introduced into *Harwich* males, an inducer of HD, its effect on the phenotype caused by HD remains elusive. Because almost all *loki*- progeny cultured at 30°C died during larval and pupal development, progeny of control, *loki*-, *Myc* KD^{RNAi1}, and *Myc* KD^{RNAi1} + *loki*- were cultured at 25°C. Gonads were obtained from adults 3–5 days after eclosion, and their morphologies were observed. Ovaries with more than three mature eggs and testes > 1 mm in length were considered normally developed gonads. Significance was calculated by two-sided Fisher's exact test; ns, not significant ($P \geq 0.05$). N: total number of the observed gonads. Similar results were obtained from two biologically independent experiments.

Supplementary Table 1. Mating scheme for examining *sn^w*-mutation frequency in the germline

Genotypes of parents	Genotypes of progeny	Abbreviations for progeny*
♀ sn^w/+; *nos-Gal4*/*TM3*, *Ser Sb*<br>♂ *y w*	sn^w/Y; *nos-Gal4*/+	Control-sn^w
♀ sn^w/+; *nos-Gal4*/*TM3*, *Ser Sb*<br>♂ *Harwich*	sn^w/Y; *nos-Gal4*/+	HD-sn^w
♀ sn^w/+; *nos-Gal4*, *UASt-Myc*/*TM3*, *Ser Sb*<br>♂ *Harwich*	sn^w/Y; *nos-Gal4*, *UASt-Myc*/+	HD-sn^w + *Myc* o/e

*Control-sn^w, HD-sn^w, and HD-sn^w + *Myc* o/e males were mated with attached-X females (XX/Y), and male offspring with the sn^w, sn^e, and sn^+ phenotypes were counted.

Supplementary Table 2. Mating scheme for examining percentage of offspring developing to adulthood

Genotypes of parents	Genotypes of progeny	Abbreviation for progeny*
♀ *nos-Gal4*/*nos-Gal4*<br>♂ *y w*	*nos-Gal4*/+	Control
♀ *nos-Gal4*/*nos-Gal4*<br>♂ *Harwich*	*nos-Gal4*/+	HD
♀ *nos-Gal4*, *UASt-Myc*/*TM3*, *Ser Sb*<br>♂ *Harwich*	*nos-Gal4*, *UASt-Myc*/+	HD + *Myc* o/e

*Control, HD, and HD + *Myc* o/e males and females were mated with *y w* females and males, respectively, and the percentages of their offspring that developed to adulthood were determined.

Supplementary Table 3. Mating scheme for examining *sn*^{w}-mutation frequency in the germline under non-HD condition

Genotypes of parents	Genotypes of progeny	Abbreviations for progeny*
♀ *sn*^{w}/+; *nos-Gal4*/*TM3*, *Ser Sb*<br>♂ *y w*	*sn*^{w}/Y; *nos-Gal4*/+	non-HD_*sn*^{w}
♀ *sn*^{w}/+; *nos-Gal4*, *UASt-Myc*/*TM3*, *Ser Sb*<br>♂ *y w*	*sn*^{w}/Y; *nos-Gal4*, *UASt-Myc*/+	non-HD_*sn*^{w} + *Myc* o/e

* non-HD_*sn*^{w} and non-HD_*sn*^{w} + *Myc* o/e males were mated with attached-X females (XX/Y), and male offspring with the *sn*^{w}, *sn*^{e}, and *sn*$^{+}$ phenotypes were counted.

Supplementary Table 4. Mating scheme for examining percentage of offspring developing to adulthood under non-HD condition

Genotypes of parents	Genotypes of progeny	Abbreviation for progeny*
♀ *nos-Gal4*/*nos-Gal4*<br>♂ *y w*	*nos-Gal4*/+	Control
♀ *nos-Gal4*, *UASt-Myc*/*TM3*, *Ser Sb*<br>♂ *y w*	*nos-Gal4*, *UASt-Myc*/+	*Myc* o/e

*Control and *Myc* o/e males and females were mated with *y w* females and males, respectively, and the percentages of their offspring that developed to adulthood were determined.
