# Supplementary material for: Myc plays an important role in Drosophila P-M hybrid dysgenesis to eliminate germline cells with genetic damage
Source: Commun Biol. 2020 Apr 22;3:185. doi: 10.1038/s42003-020-0923-3 (PMC7176646; doi:10.1038/s42003-020-0923-3)
Supplement: Supplementary file 3 — Description of Additional Supplementary Files [file 42003_2020_923_MOESM3_ESM.pdf]

Description of Additional Supplementary Files

File Name: **Supplementary Data 1**
Description: Data for figures used in this study. Each table corresponds to each Figure.
