# Supplementary material for: Myc plays an important role in Drosophila P-M hybrid dysgenesis to eliminate germline cells with genetic damage
Source: Commun Biol. 2020 Apr 22;3:185. doi: 10.1038/s42003-020-0923-3 (PMC7176646; doi:10.1038/s42003-020-0923-3)
Supplement: Supplementary file 4 — Reporting Summary [file 42003_2020_923_MOESM4_ESM.pdf]

nature research

Corresponding author(s): Satoru Kobayashi

Last updated by author(s): Mar 18, 2020

Reporting Summary

Nature Research wishes to improve the reproducibility of the work that we publish. This form provides structure for consistency and transparency in reporting. For further information on Nature Research policies, see Authors & Referees and the Editorial Policy Checklist.

Statistics

For all statistical analyses, confirm that the following items are present in the figure legend, table legend, main text, or Methods section.

n/a	Confirmed	
☐	☒	The exact sample size (*n*) for each experimental group/condition, given as a discrete number and unit of measurement
☐	☒	A statement on whether measurements were taken from distinct samples or whether the same sample was measured repeatedly
☐	☒	The statistical test(s) used AND whether they are one- or two-sided<br>*Only common tests should be described solely by name; describe more complex techniques in the Methods section.*
☒	☐	A description of all covariates tested
☐	☒	A description of any assumptions or corrections, such as tests of normality and adjustment for multiple comparisons
☐	☒	A full description of the statistical parameters including central tendency (e.g. means) or other basic estimates (e.g. regression coefficient) AND variation (e.g. standard deviation) or associated estimates of uncertainty (e.g. confidence intervals)
☐	☒	For null hypothesis testing, the test statistic (e.g. *F*, *t*, *r*) with confidence intervals, effect sizes, degrees of freedom and *P* value noted<br>*Give P values as exact values whenever suitable.*
☒	☐	For Bayesian analysis, information on the choice of priors and Markov chain Monte Carlo settings
☒	☐	For hierarchical and complex designs, identification of the appropriate level for tests and full reporting of outcomes
☒	☐	Estimates of effect sizes (e.g. Cohen's *d*, Pearson's *r*), indicating how they were calculated

Our web collection on statistics for biologists contains articles on many of the points above.

Software and code

Policy information about availability of computer code

Data collection	All immunofluorescence staining images was acquired by using Leica Application Suite software and processed by Adobe Photoshop CC or FIJI software.
Data analysis	The FIJI software was used to measure signal intensity of Myc-GFP, Myc-GFP mRNA, and Myc mRNA in the germline.

For manuscripts utilizing custom algorithms or software that are central to the research but not yet described in published literature, software must be made available to editors/reviewers. We strongly encourage code deposition in a community repository (e.g. GitHub). See the Nature Research guidelines for submitting code & software for further information.

Data

Policy information about availability of data

All manuscripts must include a data availability statement. This statement should provide the following information, where applicable:

- Accession codes, unique identifiers, or web links for publicly available datasets
- A list of figures that have associated raw data
- A description of any restrictions on data availability

All source data underlying the bargraphs and box plot presented in the figures are reported in Supplementary Data 1. All data and materials produced by this study are available from the corresponding author upon request.

Field-specific reporting

Please select the one below that is the best fit for your research. If you are not sure, read the appropriate sections before making your selection.

☒ Life sciences ☐ Behavioural & social sciences ☐ Ecological, evolutionary & environmental sciences

For a reference copy of the document with all sections, see nature.com/documents/nr-reporting-summary-flat.pdf

Life sciences study design

All studies must disclose on these points even when the disclosure is negative.

Sample size	Sample sizes were determined based on published studies in this field or our published data. No statistics was used to predetermine the sample size.
Data exclusions	No data were excluded intentionally.
Replication	All attempts at replication were successful, except for Fig. S8c. The percentage of pH3-positive germline cells was slightly downregulated (Experiment 1) or upregurated (Experiment 2) by Myc KD. The data combining these two results and statistical significance are shown in Fig. 8c. The data for Myc KD + p53- in Fig. S10 was obtained from a single experiment, due to a failure in maintaining Myc KD + p53- line.
Randomization	Randomly collected embryos, larvae, and flies were subjected in each experiment.
Blinding	Investigator was not blinded to fly genotypes during experiments. However, because embryos, larvae, and flies were randomly collected, the investigator was unaware of the outcome of the experiments. Thus, the investigator was totally blind for the data sampling.

Reporting for specific materials, systems and methods

We require information from authors about some types of materials, experimental systems and methods used in many studies. Here, indicate whether each material, system or method listed is relevant to your study. If you are not sure if a list item applies to your research, read the appropriate section before selecting a response.

Materials & experimental systems

n/a	Involved in the study	
☐	☒	Antibodies
☒	☐	Eukaryotic cell lines
☒	☐	Palaeontology
☐	☒	Animals and other organisms
☒	☐	Human research participants
☒	☐	Clinical data

Methods

n/a	Involved in the study	
☒	☐	ChIP-seq
☒	☐	Flow cytometry
☒	☐	MRI-based neuroimaging

Antibodies

Antibodies used	Information on all antibodies used in this study was provided in methods section.
Validation	anti-Vasa antibody. Validation: Hayashi et al., Sci. Rep. 2017<br>anti-Hts antibody. Validation: http://dshb.biology.uiowa.edu/adducin-related-protein<br>anti-Fasciclin III antibody. Validation: http://dshb.biology.uiowa.edu/7G10-anti-Fasciclin-III_2<br>anti-GFP antibody. Validation: https://www.thermofisher.com/antibody/product/GFP-Antibody-Polyclonal/A-11122<br>anti-phospho-Histone H3 antibody. Validation: http://www.merckmillipore.com/JP/ja/product/Anti-phospho-Histone-H3-Ser10-Antibody-Mitosis-Marker,MM_NF-06-570<br>horseradish peroxidase–conjugated anti-DIG antibody. Validation: https://www.sigmaaldrich.com/catalog/product/roche/11207733910?lang=en&region=US

Animals and other organisms

Policy information about studies involving animals; ARRIVE guidelines recommended for reporting animal research

Laboratory animals	Information on all fly strains used in this study was provided in methods section.
Wild animals	This study did not use wild animals.
Field-collected samples	This study did not use samples collected from the field.

Ethics oversight	Not applicable

Note that full information on the approval of the study protocol must also be provided in the manuscript.
